# Attenuation of a Slow Subsonic A_0_ Mode Ultrasonic Guided Wave in Thin Plastic Films

**DOI:** 10.3390/ma12101648

**Published:** 2019-05-21

**Authors:** Rymantas Kažys, Reimondas Šliteris, Liudas Mažeika, Olgirdas Tumšys, Egidijus Žukauskas

**Affiliations:** Ultrasound Institute, Kaunas University of Technology, Baršausko str. 59, LT-51368 Kaunas, Lithuania; reimondas.sliteris@ktu.lt (R.Š.); liudas.mazeika@ktu.lt (L.M.); olgirdas.tumsys@ktu.lt (O.T.); e.zukauskas@ktu.lt (E.Ž.)

**Keywords:** ultrasonic guided waves, subsonic A_0_ Lamb wave mode, attenuation of subsonic A_0_ mode

## Abstract

The ultrasonic testing technique using Lamb waves is widely used for the non-destructive testing and evaluation of various structures. For air-coupled excitation and the reception of A_0_ mode Lamb waves, leaky guided waves are usually exploited. However, at low frequencies (<100 kHz), the velocity of this mode in plastic and composite materials can become slower than the ultrasound velocity in air, and its propagation in films is accompanied only by an evanescent wave in air. To date, the information about the attenuation of the slow A_0_ mode is very contradictory. Therefore, the objective of this investigation was the measurement of the attenuation of the slow A_0_ mode in thin plastic films. The measurement of the attenuation of normal displacements of the film caused by a propagating slow A_0_ mode is discussed. The normal displacements of the film at different distances from the source were measured by a laser interferometer. In order to reduce diffraction errors, the measurement method based on the excitation of cylindrical but not plane waves was proposed. The slow A_0_ mode was excited in the polyvinylchloride film by a dry contact type ultrasonic transducer made of high-efficiency PMN-32%PT strip-like piezoelectric crystal. It was found that that the attenuation of the slow A_0_ mode in PVC film at the frequency of 44 kHz is 2 dB/cm. The obtained results can be useful for the development of quality control methods for plastic films.

## 1. Introduction

Non-destructive testing (NDT) methods can reduce the possibility of the failure of manufactured products and fill the need for their improved quality. Many different methods of NDT, such as resonant inspection, ultrasonics, radiography, eddy currents, liquid penetrants, magnetic particles and infrared thermography [1,2,3,4,5], are now in standard use in civil, aeronautical and systems engineering. However, most of these—except ultrasonic technique—are not suitable for the inspection of the elastic properties of thin plastic films. The work described here aims to determine which ultrasound technology can be applied to the NDT of thin plates using ultrasonic guided waves. 

Ultrasonic guided Lamb waves are already widely used for the non-destructive testing and evaluation of pipes, thin plates and films [6,7,8,9,10,11,12,13]. Their propagation characteristics are sensitive to structural changes in the material through which they travel. Compared with other waves, Lamb waves can propagate longer distances with minimal loss of energy and low amplitude attenuation. The Lamb waves are dispersive, which leads to the presence of multiple modes, and as a consequence, their velocity and attenuation depend on the frequency and the type of the guided wave [13]. In order to avoid the presence of multiple modes in the signals, and in such a way as to simplify signal processing, only the lowest fundamental guided wave modes such as symmetrical S_0_, anti-symmetrical A_0_, shear horizontal SH_0_ and torsional waves are usually used in non-destructive testing applications. One of the most important parameters from the perspective of long-range ultrasonic testing is the losses of a particular guided wave mode. The losses are caused by the attenuation of the guided wave due to material properties and due to the radiation of a leaky wave into the surrounding medium, which in the case of surrounding liquids or a soil may be significant.

The attenuation of low-frequency guided waves has mainly been investigated for the waves used for long-range ultrasonic inspection purposes. Most works have been devoted to the analysis of torsional and longitudinal waves because they are least attenuated when the structure under inspection is loaded by fluid or soil.

The theoretical analysis of the propagation of guided waves in absorbing and non-absorbing plates was performed in [14], but the main attention was paid to the energy velocity. 

The theoretical analysis of leaky guided waves in fluid-loaded waveguides using the semi-analytical finite element approach was presented in [15], but the results were analysed only for a titanium circular bar and square and L-shaped steel bars immersed in water.

The attenuation of torsional guided waves in pipes buried in soil at low frequencies (<30 kHz) was measured in [16]. The attenuation of guided torsional and longitudinal modes in pipes buried in sand in the frequency range 11–34 kHz was also investigated by [17]. It was shown that significant losses of guided waves are due to energy leaking into the imbedding soil. The attenuation of guided waves in carbon fibre-reinforced composites was analysed in [18].

The above-mentioned waves were excited and picked up by contact methods. The inspection of thin plates and plastic films may be efficiently performed by ultrasonic air-coupled non-destructive evaluation methods exploiting guided waves [19,20,21,22,23,24]. In the case of air-coupled excitation, usually an A_0_ Lamb wave mode is used, which is sensitive to various non-homogeneities in the object under testing. For air-coupled excitation and the reception of the A_0_ mode, leaky guided waves are usually exploited. However, at low frequencies (<100 kHz), the velocity of this mode in plastic and composite materials can become slower than the ultrasound velocity in air, and its propagation in films is accompanied only by an evanescent wave in air. Such a mode is called a subsonic [13] or a slow A_0_ mode [25]. Taking into account the fact that there is no leaky wave to excite or pick-up such a wave by a conventional air-coupled technique, using the ultrasonic transducers deflected according to Snell’s law is impossible; however, this can be done by a properly phased multi- element linear array [25].

In this case, the A_0_ mode in the subsonic regime, when the phased velocity of the A_0_ mode $c_{{\mathrm{phA}}_{0}}$ is slower than the velocity in air $c_{\mathrm{air}}$, e.g., $c_{{\mathrm{phA}}_{0}}<c_{\mathrm{air}}$, is trapped in the plate and causes only an evanescent wave in the surrounding air whose amplitude strongly decreases with the increase of the distance from the plate under investigation [26,27]. There is no leaky wave caused by a slow A_0_ mode, and consequently no attenuation due to energy leaks into the surrounding medium. Due to this fact, the total propagation losses should be lower and it should be attractive for non-destructive evaluation purposes. However, until now information about attenuation of the slow A_0_ mode is very contradictory. Some studies were devoted to the analysis of the propagation of such guided waves in plates loaded by some fluid; for example, water. In this case, A mode splitting is observed [28] and, according to the dispersion equation, the interface wave called a Scholte or quasi-Scholte wave propagates in a fluid along the fluid–film interface [13,26,27]. The attenuation in this case is very dependent on frequency and can vary from almost zero (Scholte waves or slow A_0_ mode at low frequencies) up to hundreds of Np/m for the fast A_0_ mode [13,26].

In the case of plastic films placed in air, the loading of the film is negligible, and at low frequencies, only the slow subsonic A_0_ mode propagates in plastic films. In fact, no data regarding the attenuation of this mode in unloaded or placed in air plastic films could be found.

Therefore, the objective of this investigation was the measurement of the attenuation of the slow A_0_ mode in thin plastic films. The polyvinylchloride (PVC) film (135 µm) which is widely used for packaging purposes was selected for the investigation. The production of PVC films in Europe alone reaches 5 million tons annually, and the non-destructive evaluation of the quality of such films is very important [29].

The paper is organized as follows. In Section 2, the measurement method of the attenuation coefficient is proposed and theoretically analysed. In Section 3, the experimental set-up and measurement procedures are described. In Section 4, the measurement results are presented. In Section 5, the conclusions drawn from the theoretical and experimental results are presented.

## 2. Theoretical Analysis

### 2.1. Theoretical Background for the Measurement of the Attenuation

**Figure 1:**
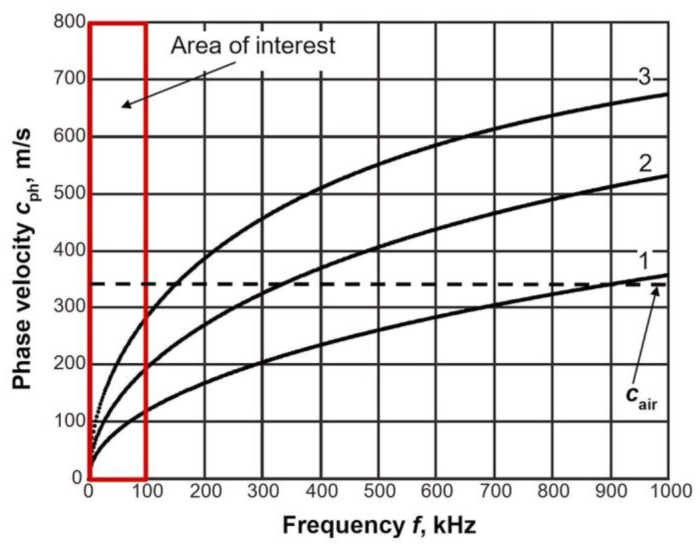
The dispersion curves of the Lamb wave A_0_ mode in PVC films of different thicknesses *d*: 1—*d* = 0.05 mm; 2—*d* = 0.135 mm; 3—*d* = 0.3 mm. The dashed line indicates the ultrasound velocity in air (*c*_air_ = 342 m/s).

The phase and group velocities of the A_0_ mode guided wave are frequency-dependent. Dependencies of those velocities versus frequency are called dispersion curves. The dispersion curves of the A_0_ Lamb wave mode phase velocity in PVC films of different thicknesses were calculated using the semi analytical finite element (SAFE) method and are presented in Figure 1. The calculations were performed using the following parameters of the PVC film [30]: the Young‘s modulus *E* = 2.937 GPa; the Poisson coefficient *ν* = 0.42; the density *ρ* = 1400 kg/m^3^; and the thickness of the plate *d* = 50–300 μm. The film was split into a finite number of thin layers, each of which is described in the one axis direction by three nodes, and in the second axis direction, it is assumed that the plate is infinite [6].

From the results presented in Figure 1, it follows that in a quite wide range of frequencies (<900 kHz), the phase velocity of A_0_ mode in PVC films is lower than the ultrasound velocity in air *c*_air_ = 342 m/s. The low- frequency range in which the slow Lamb wave A_0_ mode was generated is shown in Figure 1 by the rectangle. At those frequencies, the propagating A_0_ mode is called a slow or sub-sonic wave [13]. In this case, the wave is trapped inside the film and accompanied not by a leaky but an evanescent wave in air. This means that the attenuation of such a slow A_0_ mode should be only due to losses in the plastic material.

Usually, in the case of the air-coupled ultrasonic non-destructive evaluation, the reception of guided waves leaky waves in air is exploited. This is created only by normal displacements of a film under a test. Therefore, we shall further discuss the measurement of the attenuation of normal displacements $\xi (x_{j},t)$ caused by the propagating slow A_0_ mode. Here, *x_j_* is the spatial coordinate and *t* is the time.

Usually, the attenuation of an ultrasonic wave is obtained from the amplitudes of a plane ultrasonic wave measured at two different distances. Application of the plane wave allows us to avoid measurement errors due to diffraction. However, in our case, at frequencies lower than 50 kHz, to excite a plane A_0_ mode wave in the film is unrealistic.

Therefore, for attenuation measurement, instead of a plane wave, we proposed the use of a cylindrical wave excited by a point type source. As such a source, a transducer creating displacements normal to the film with lateral dimensions smaller than wavelength of A_0_ mode can be used. In this case, the normal displacement $\xi (x_{j},t)$ of the film at the distance *x_j_* from the source is given by [13]

(1) $$\xi \left(x_{j},t\right)=\frac{\xi_{0}}{\sqrt{x_{j}}}e^{-i\left(2\pi ft-kx_{j}\right)}e^{-\alpha \left(f\right)x_{j}},$$

where *t* is the time, *ξ*_0_ is the normal displacement at the centre of the point type source, *k* is the wave number, and α(*f*) is the frequency *f*-dependent attenuation coefficient.

The normal displacements of the film at different distances from the source can be measured by a laser interferometer. Such measurements allow the avoidance of the influence of the ultrasonic wave propagating from the excitation source in air.

The spectrum of the normal displacement at the distance *x*_j_ is given by

(2) $$S_{\xi }\left(x_{j},t\right)=\mathrm{FFT}\left[\xi \left(x_{j},t\right)\right]=\frac{\xi_{0}}{\sqrt{x_{j}}}e^{-\alpha \left(f\right)x_{j}}e^{-ikx_{j}},$$

where FFT is the fast Fourier transform. From spectra obtained at different distances at selected frequencies, it is possible to obtain the attenuation coefficient *α*(*f*):

(3) $$\alpha \left(f\right)=\frac{1}{x_{n}-x_{m}}\ln\left(\frac{\left|S_{m}\left(x_{m},f\right)\right|\cdot \sqrt{x_{m}}}{\left|S_{n}\left(x_{n},f\right)\right|\cdot \sqrt{x_{n}}}\right),$$

where $S_{m}(x_{m},f)$ and $S_{n}(x_{n},f)$ are the spectra of the normal displacements at the distances *x_m_* and *x_n_*, respectively.

Another way to obtain the attenuation coefficient *α* (*f*) is based on approximation of the measured normal displacement dependence versus distance $\hat{\xi }(x_{j})$ by the theoretical dependence given by Equation (1):

(4) $$\xi \left(x_{j}\right)=\frac{\xi_{0}}{\sqrt{x_{j}}}e^{-\alpha \left(f\right)x_{j}},$$

During approximation, the attenuation coefficient *α*(*f*) in Equation (4) is modified until the best fit to the experimental results is obtained. Such a procedure can be performed by the Nelder–Mead simplex algorithm [31], which enables us to find the minimum of the unconstrained multivariable function using a derivative-free method:

(5) $$M=\underset{\alpha \left(f\right)=\hat{\alpha }\left(f\right)}{\min}\sum_{j=1}^{J}\left|\xi \left(x_{j}\right)-\overset{⌢}{\xi }\left(x_{j}\right)\right|,$$

where *J* is the total number of the measurement points. The value of the attenuation coefficient $\hat{\alpha }(f)$ at which the minimum of the function in Equation (5) is obtained corresponds to the measured attenuation coefficient of the A_0_ mode in the film.

This approach should be more accurate than the method based on measurement at two different distances because, in the latter case, the measurement result is obtained from many amplitude measurements at different distances *x_j_* from the source.

The accuracy of such a method first of all depends on how close the used source of the A_0_ mode is to a point type source. If this requirement is not fulfilled, then the decay of the normal displacement amplitude along the distance from the source cannot be described by Equations (1) and (2), which will introduce additional diffraction errors into the results of measurements. From a practical point of a view, any kind of source possesses finite dimensions. For the excitation of the A_0_ mode, a contact-type transducer with a high efficiency PMN-32%PT strip-like piezoelectric crystal operating in a longitudinal-extension mode has been used [32]. This transducer is described in more detail in Section 3. The exciting aperture is a rectangular tip of the transducer with dimensions of 1 × 5 mm^2^. In order to check how close the aperture is to a point type source at the operation frequency of 44 kHz, finite element modelling of the excitation and propagation of the guided wave in a PVC film was performed.

### 2.2. Finite Element Modelling

**Figure 2:**
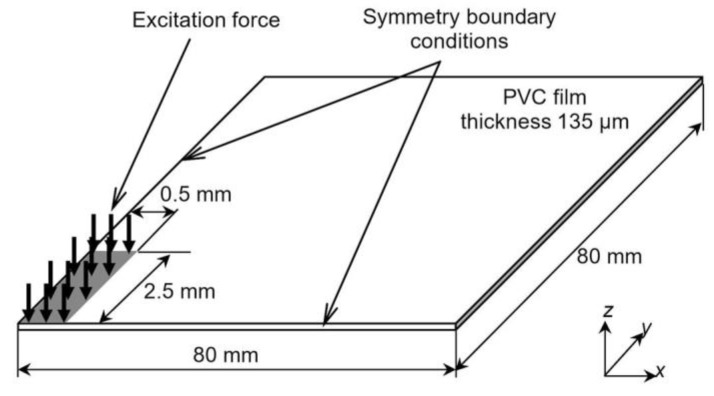
Graphical representation of the finite element model (dimensions are not to scale).

The numerical investigation of the propagation of a pulsed ultrasonic A_0_ Lamb wave in the 135-µm thick 3D PVC film was carried out using the Abaqus 6.16 finite element software. To solve the transient wave equation, an explicit algorithm was used. In order to reduce the computational time, only a quarter of the plate with symmetry boundary conditions was modelled. A graphical representation of the model is presented in Figure 2.

The modelled film is placed in a vacuum. The attenuation coefficient of the A_0_ mode in the material was assumed to be negligible.

**Figure 3:**
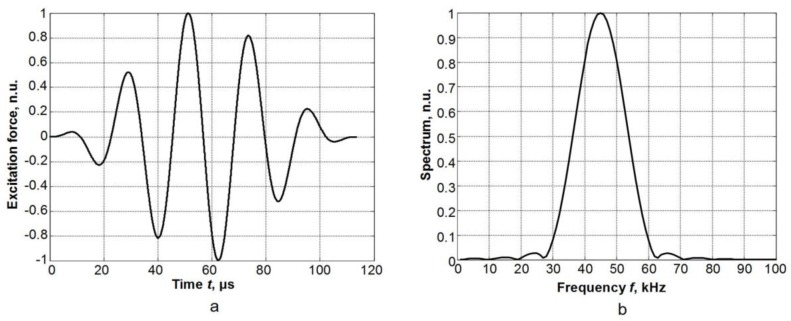
Time diagram (**a**) and spectrum (**b**) of the excitation force; n.u.—normalised unit.

In order to excite the asymmetric A_0_ guided wave in the PVC film, the normal transient excitation force of 1 N was applied to the selected nodes on the ultrasonic transducer zone. The modelling was performed using two different dimensions of the excitation zone: a 1 × 1 mm^2^ zone that corresponds to a point type source, and a 1 × 5 mm^2^ zone which corresponds to the dimensions of the radiating aperture used in the experimental investigation. The excitation force is uniformly distributed on the excitation surface. The excitation force was of 44 kHz 5 periods burst with the Gaussian envelope. The frequency was chosen to be the same as the operation frequency of the high-efficiency ultrasonic transducer used in the experimental investigations. The time diagram and the spectrum of a transient excitation force are presented in Figure 3a,b.

The modelling of wave propagation was performed by solving the following dynamic equation:

(6) $$\left[M\right]\left\{\ddot{U}\right\}+\left[C\right]\left\{\dot{U}\right\}+\left[K\right]\left\{U\right\}=\left\{F(t)\right\},$$

where [**M**] is the structural mass matrix, [**C**] is the element damping matrix, [**K**] is the structural stiffness matrix, {**U**} is the displacement vector, and {**F**} is the structural load vector. The Abaqus explicit software uses a central difference method to solve the dynamic equation through time.

The 3D geometry of the PVC film was meshed using 8 nodes and linear brick elements of C3D8R with a hourglass control. The hourglass control prevents the propagation of zero energy modes through the mesh, which may lead to inaccurate solutions. The size of the finite elements was 65 µm. This size corresponds to 1/45^th^ of the wavelength of the slowest A_0_ mode ultrasonic Lamb wave at the frequency of 44 kHz. The central difference integration method is conditionally stable, and the most critical variable using this method is the time step size Δ*t*. The time step Δ*t* must be smaller than the stability limit of the central difference method. If the time step size is not small enough, then the solution becomes unstable. In our case, the stable solutions are obtained with a time step duration of 10 ns.

**Figure 4:**
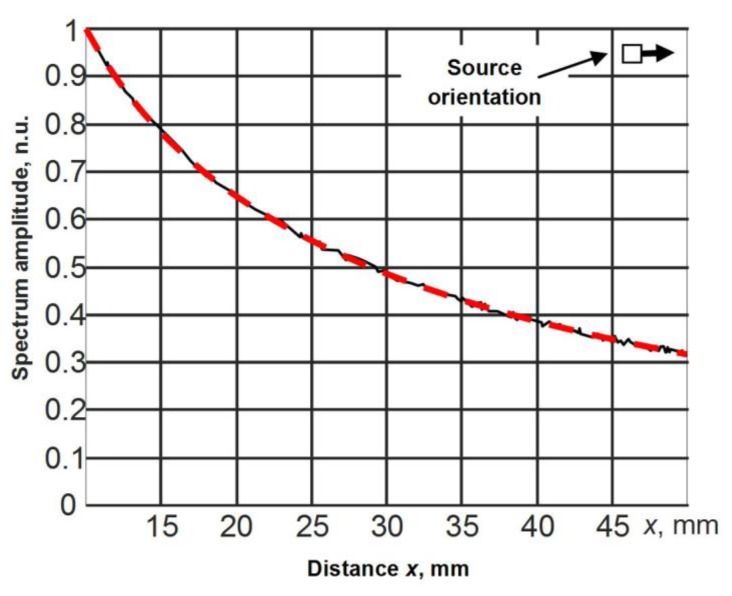
Dependence of spectrum amplitude at 44 kHz on the distance from the point type 1 × 1 mm^2^ excitation point. Solid line—finite element modelling data, dashed line—curve fitting.

In the case of the point type excitation, and when the attenuation in the material is neglected, the amplitude of the propagating guided wave should decrease only due to the diffraction effect according to the 1/$\sqrt{r}$ law, where *r* is the distance from the excitation source. In order to check this, the calculated normal displacements at each node along the *x* axis were recorded, the spectra of all signals were calculated and the spectra values at the frequency of 44 kHz were extracted. The result is presented in Figure 4 by the solid line. The normal displacement amplitudes versus distance were approximated according to Equations (4) and (5) by the above-described Nelder–Mead simplex algorithm. The obtained result is shown in Figure 4 by the dashed line. It was found that, in this case, 1/$\sqrt{r}$ curve-fitting revealed the presence of an additional 0.75 dB/cm attenuation (Figure 4). This phenomenon can be explained by a numerical attenuation introduced by the finite element software, and therefore should be neglected as a numerical artefact. In the Abaqus explicit software, a small amount of numerical damping is introduced by default in a form of bulk viscosity to control high-frequency oscillations [33].

**Figure 5:**
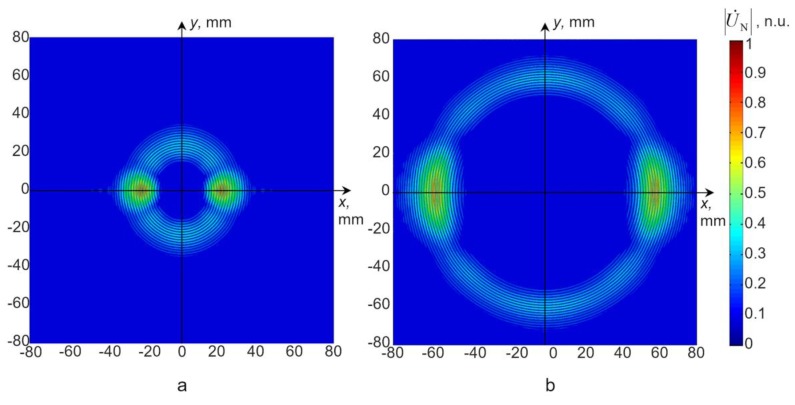
Normalized modulus of particle velocity at different instances of time in the case of a 1 × 5 mm^2^ excitation zone. (**a**) 150 µs, (**b**) 300 µs.

**Figure 6:**
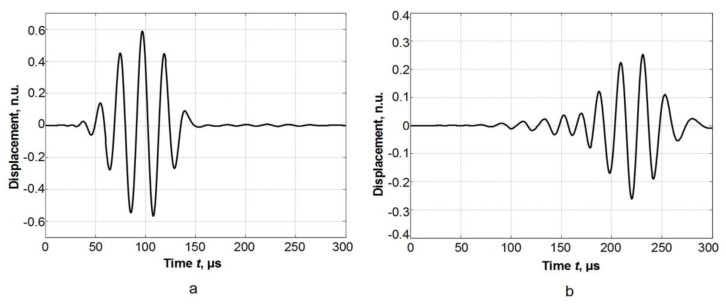
A-scans along the x axis at different distances from the excitation zone. (**a**) At a distance of 10 mm, (**b**) at a distance of 45 mm.

**Figure 7:**
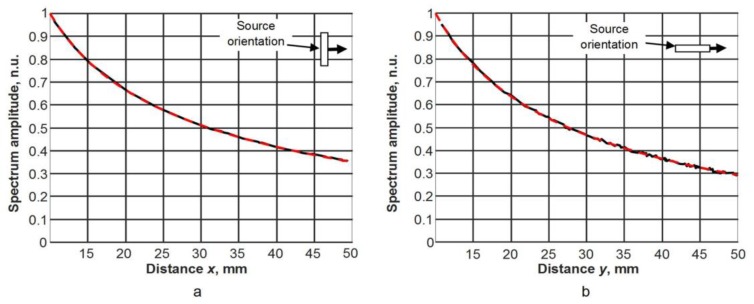
Dependence of spectrum amplitudes at 44 kHz on the distance from the excitation zone. (**a**) Results obtained along the *x* axis, (**b**) results obtained along the *y* axis. Solid line—finite element modelling data, dashed line—curve fitting.

In the case of the 1 × 5 mm^2^ size excitation zone, a modulus of the particle velocity field at two different time instants is shown in Figure 5. Spatial distributions of the particle velocity field were analysed along *x* and *y* symmetry axes (see Figure 2). A-scans at different distances from the excitation zone along the *x* axis are presented in Figure 6. The signal amplitudes were normalized according to the amplitude maximum of the signal at the excitation zone. An approximation of the calculated dependences was performed in the same way as in the case of the point type source. An analysis of the signal spectra along *x* and *y* axes revealed that amplitude decay law is very close to the results obtained for a point type excitation (Figure 7a,b). In the case of the signals obtained along the *x* axis, the additional numerical attenuation is 0.52 dB/cm, and in the case of the signals along the *y* axis, the additional numerical attenuation is 0.7 dB/cm. Both those numerical attenuations may be neglected as before because no material attenuation was taken into account during finite element modelling. The obtained results allow us to make the assumption that a 1 × 5 mm^2^ size ultrasonic transducer at 44 kHz can be analysed in the same way as a point type transducer, and for the A_0_ mode attenuation measurement, it is possible to use the method presented above (Equations (4) and (5)).

## 3. Experimental Set-Up

**Figure 8:**
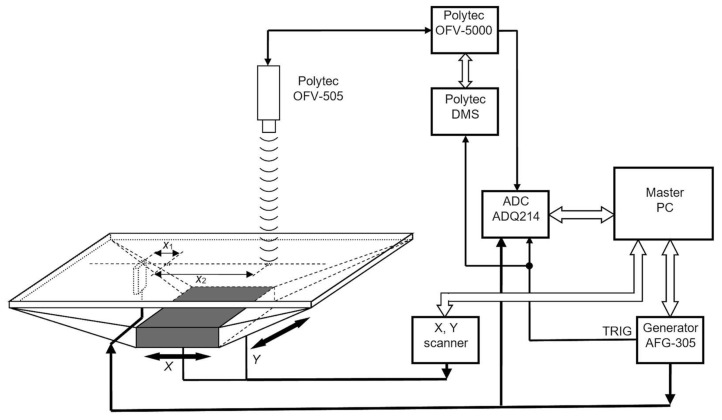
The experimental set-up for the measurement of A_0_ mode Lamb wave attenuation in a PVC film.

For measurements of the attenuation of a sub-sonic A_0_ Lamb wave mode, a thin polyvinyl chloride (PVC, London, UK) film was chosen. The film with lateral dimensions 210 × 297 mm^2^ and 0.135 mm thickness was fixed in a specially made rectangular frame (Figure 8). For the excitation of the A_0_ Lamb wave mode, a rectangular strip-like piezoelectric element with dimensions of 15 × 5 × 1 mm^3^ made of the PMN-32%PT piezoelectric crystal (HC Materials Corporation, Bolingbrook, IL, USA) was exploited. The piezoelectric element was excited in the transverse-extension mode at the lowest resonance frequency *f*_0_ = 44 kHz. Regarding radiation, the tip of the piezoelectric strip with a rectangular aperture of 5 × 1 mm^2^ was used. In this case, the sides of the radiating aperture *a* and *b* are close to or less than the wavelength λ_A_0__ of the A_0_ mode in the film; e.g., *a*/λ_A_0__ = 1.58 and *b*/λ_A_0__ = 0.32. This means that such a source of ultrasonic waves may be considered as close to a point type source, and correspondingly, attenuation values measured along directions *x* and *y* in the case of an isotropic material should be close to each other.

The used strip-like PMN-32%PT [34] piezoelectric elements in a transverse-extension mode possess a very high electromechanical coupling coefficient *k*_32_ (0.84–0.90), which makes them attractive for low-frequency applications [31]. To improve the bandwidth and efficiency of radiation, a quarter wavelength matching strip made of AIREX T90.210 [35] type polystyrene foam (AIREX AG, Sins, Switzerland) was bonded to the tip of the piezoelectric strip. The excitation of A_0_ Lamb wave was performed via a dry contact between the matching strip and the film.

For attenuation measurements, normal displacements of the film versus distances along *x* and *y* directions perpendicular to the long and short sides of the rectangular aperture 5 × 1 mm^2^ were measured by the Polytec laser interferometer OFV-5000 (Polytec GmbH, Waldbronn, Germany) [36] (Figure 8). For this purpose, the supporting frame and the PVC film were scanned together with the dry-contact ultrasonic transducer with respect to the laser beam. This allowed us to achieve a stable acoustic contact during measurements between the strip-like ultrasonic transducer and the film. Two-dimensional scanning along *x* and *y* directions was performed by the 2D scanner 8MTF-75LS05 [37] (Standa, Vilnius, Lithuania) (Figure 8).

The ultrasonic transducer was excited by AFG-3051 [38] (GW INSTEC, Taiwan) generator using the 70-cycle duration electric pulse with a 0.5 V amplitude and with a 0.5 s repetition period. The used ultrasonic transducer is relatively narrowband; therefore, in order to achieve the steady state amplitude of vibrations and to increase the accuracy of measurements, 70-cycle excitation is required. The normal displacement waveforms of the film registered by the Polytec laser interferometer were digitized and recorded by the analog–digital converter ADQ214 [39] (Teledyne SP Devices, Vaxholm, Sweden) with a sampling frequency of 50 MHz. In order to improve the signal to noise ratio at each measurement point, 5–10 signals were averaged. The synchronization of the whole measurement system and saving of recorded signals is performed by a master computer.

**Figure 9:**
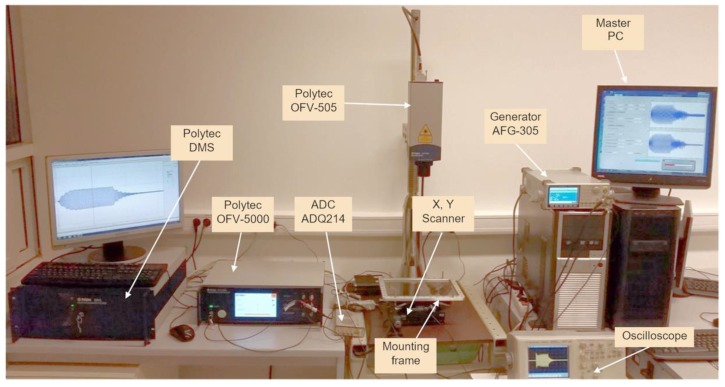
The overall view of experimental set-up.

**Figure 10:**
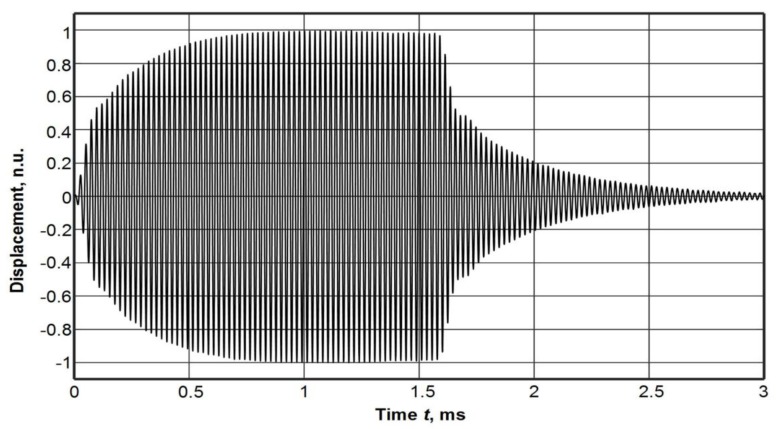
The normal displacement signal of the PVC film measured above the centre of the transducer radiating surface.

The view of the experimental set-up is shown in Figure 9. An example of the normal displacement signal of a thin film measured above the centre of the ultrasonic transducer aperture is shown in Figure 10.

## 4. Experimental Results

The measurement of the attenuation of the A_0_ mode Lamb wave was based on the collection of waveforms of normal displacement signals $\xi (x_{j},y_{i},t)$ at different distances from the exciting ultrasonic transducer and comparing them with calculated amplitude versus distance dependences made under the assumption that the wave is excited by a point type source. Such measurements were performed at two orthogonal directions, *x* and *y*, which are perpendicular to the longer and shorter sides of the radiating aperture.

The scanning of the frame with the attached ultrasonic transducer was performed from 10 mm to 80 mm between the incident laser beam and the ultrasonic transducer. The 0 mm distance corresponds to the centre of the radiating aperture.

**Figure 11:**
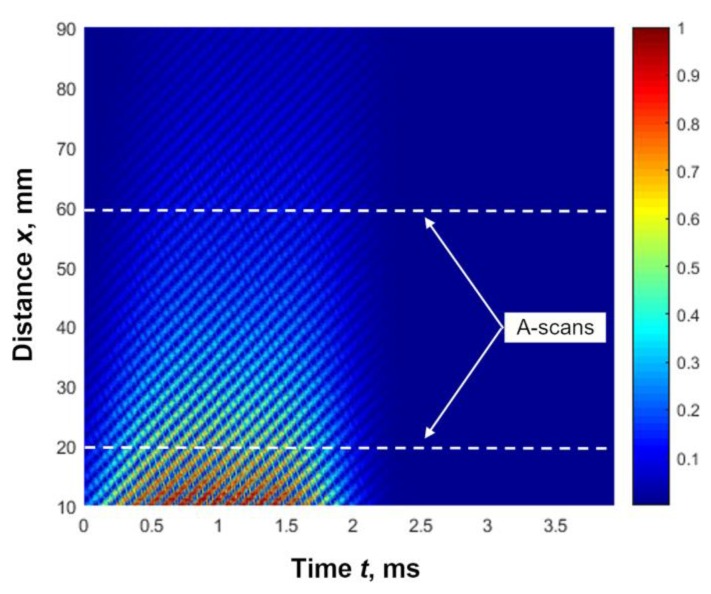
Measured B-scan of the normal displacement of the PVC film.

The displacement signals were registered by the laser interferometer with initial laser beam positions of *x*_1_ = 10 mm or *y*_1_ = 10 mm, depending on the measurement direction. The scanning step was 0.5 mm. All collected A-scans were normalized according to the maximum amplitudes of the A-scans at the distances *x* = 10 mm or *y* = 10 mm. The B-scan of the measured normal displacements of the PVC film is presented in Figure 11. The amplitudes of the normal displacement presented in Figure 11 by different colours were normalized with respect to the maximal amplitude obtained at the distance *x* = 10 mm. According to this B-scan, the phase velocity of the A_0_ mode in the investigated PVC film was 139 m/s; e.g., much slower than the ultrasound velocity in air.

**Figure 12:**
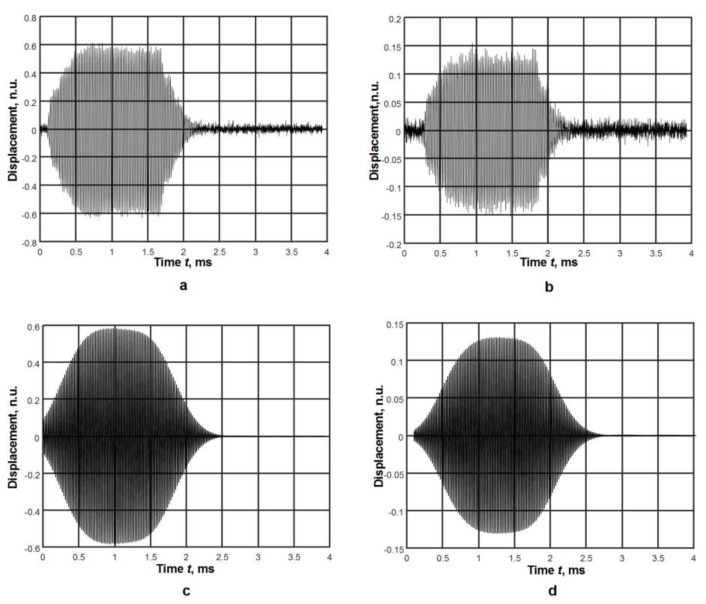
Raw (**a**) and (**b**) and filtered (**c**) and (**d**) A-scans at different distances from the piezoelectric transducer: (**a**) and (**c**) the distance is *x*_j_ = 20 mm, (**b**) and (**d**) the distance is *x*_j_ = 60 mm.

The collected raw A-scans at the distances *x*_j_ = 20 mm and *x*_j_ = 60 mm are shown in Figure 12a,b. From the presented signal, it follows that they are affected by noise. Therefore, in order to increase the accuracy of measurements, they were filtered by a bandpass Gaussian filter with the following parameters: a central frequency of 44 kHz, and a filter bandwidth of 0.9 kHz. The filtered signals are presented in Figure 12c,d.

The attenuation of the A_0_ mode is frequency-dependent; therefore, it was evaluated at a fixed selected frequency. For this purpose, the spectra of the filtered waveforms $\xi (x_{j},y_{i},t)$ were calculated using a fast Fourier transform:

(7) $$S_{\xi }\left(x_{j},y_{i},t\right)=\mathrm{FFT}\left[\xi \left(x_{j},y_{i},t\right)\right],$$

**Figure 13:**
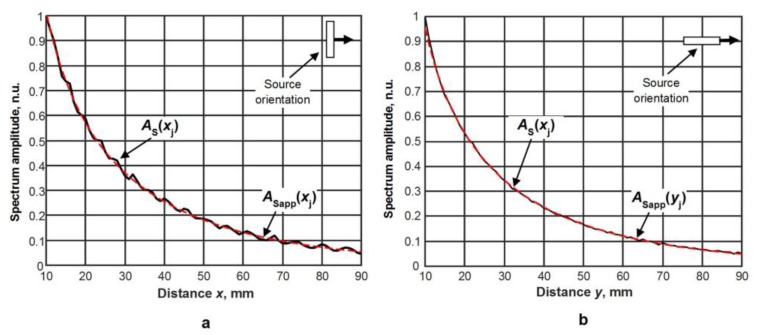
Dependencies of the maximum amplitudes $A_{S}(x_{j},y_{i})$ of the spectra of the measured signals versus distance: (**a**) wide edge (5 mm) scan direction, (**b**) narrow edge (1 mm) scan direction.

In each spectrum, at the frequency of 44 kHz, the amplitude values $A_{S}(x_{j},y_{i})$ at different distances *x_j_* and *y_i_* from the excitation aperture were found. Their dependencies versus distance are shown in Figure 13a,b by black solid lines. The amplitudes presented in Figure 13 were normalized with respect to the amplitude value at the position 10 mm. The results are presented for two orthogonal directions, *x* and *y*, corresponding to the wide and the narrow edge of the radiating aperture.

Such measurements allow us to evaluate how the used source with an aperture of 5 × 1 mm^2^ is close to the point type source. The maximum amplitudes of the spectra $A_{S}(x_{j})$ of signals versus *x* and *y* directions corresponding to different orientations of the ultrasonic transducer were approximated by expressions which are valid for a point type source:

(8) $$A_{\mathrm{Sapp}}\left(x_{j}\right)=K_{1}^{x}\frac{1}{\sqrt{x_{j}}}e^{-K_{2}^{x}x_{j}},$$

(9) $$A_{\mathrm{Sapp}}\left(x_{j}\right)=K_{1}^{y}\frac{1}{\sqrt{y_{j}}}e^{-K_{2}^{y}y_{j}},$$

In those equations, $K_{1}^{x}$ and $K_{1}^{y}$ are the normalization coefficients, and $K_{2}^{x}$ and $K_{2}^{y}$ represent the measured attenuation coefficients α(f) of the A_0_ mode Lamb wave in *x* and *y* directions. The results obtained from those approximations are shown in Figure 13 by red solid lines. There is a very good correspondence between the measurement data and the approximations. This means that the used ultrasonic transducer with a rectangular aperture can be considered as a point like source and the attenuation coefficient can be found from Equations (8) and (9). The attenuation coefficients calculated from those curves are the following: along the *x* direction, 1.99 dB/cm, and along the *y* direction, 2.03 dB/cm; e.g. the difference between the attenuation coefficients in *x* and *y* directions is only 0.04 dB/cm.

Taking into account the fact that this difference is very small, it is possible to make the conclusion that the attenuation of the slow A_0_ mode in a PVC film at the frequency of 44 kHz is 2 dB/cm.

The measurement uncertainty mainly depends on the uncertainty of normal displacement measurements performed by the laser interferometer and the uncertainties of spatial coordinates *x*_j_ and *y*_i_. The latter uncertainty is due to the 2D scanner performing scanning. The spatial resolution of it is very high; the minimal scanning step is 10 μm. This means that uncertainty due to the scanner may be neglected.

Therefore, the uncertainty of the measured normal displacement amplitudes is found to be the standard deviation of the peak amplitude of the spectrum at the selected frequency in the whole measurement range from 10 mm to 90 mm:

(10) $$\Delta A=\sqrt{\frac{1}{J-1}\sum_{j=1}^{J}{\left(A_{S}\left(x_{j}\right)-A_{\mathrm{Sapp}}\left(x_{j}\right)\right)}^{2}},$$

where *J* is the total number of the measurement points. The total number of measurements from which the uncertainty was estimated was 1500. The scattering of the measured values $A_{S}\left(x_{j}\right)$ was evaluated with respect to the approximation curve $A_{\mathrm{Sapp}}\left(x_{j}\right)$ and the maximum possible deviation of the attenuation coefficient α(f) was obtained. The uncertainties of the attenuation coefficients evaluated in such a way are the following: along the *x* direction, ±0.21 dB/cm, and along the *y* direction, ±0.16 dB/cm.

## 5. Conclusions

For the measurement of the attenuation of the slow A_0_ mode in films, the method based on the application of a point type source of a guided wave was proposed. Such an approach allows the avoidance of diffraction errors that would be impossible to eliminate in the case of measurements with quasi-planar waves at low frequencies. For the excitation of the A_0_ mode, an ultrasonic transducer made of a high-efficiency PMN-32%PT strip-like piezoelectric crystal operating in a longitudinal-extension mode was used. For radiation, the tip of the piezoelectric strip with a rectangular aperture of 5 × 1 mm^2^ was used. In this case, the sides of the radiating aperture are close or less than the wavelength λ_A_0__ of the A_0_ mode in the film at the frequency of 44 kHz, which means that such a source may be considered as close to a point type source. This was also confirmed by a finite element modelling and by measurements performed in two orthogonal directions. Measurements of the attenuation of the sub-sonic A_0_ Lamb wave mode were performed in a polyvinyl chloride (PVC) film with lateral dimensions of 210 × 297 mm^2^ and a thickness of 0.135 mm. The measured attenuation coefficient for this mode in PVC film at the frequency of 44 kHz is 2 dB/cm. The measurements at different frequencies will be correct if the assumption of the point type source for those frequencies is still valid.

It is necessary to point out that, during the manufacturing of PVC films, various defects such as wrinkles, holes, a rough surface and thickness variations arise [40]. Some of these are detected by optical methods, but such defects as holes and especially thickness variations could be found by ultrasonic methods using the discussed A_0_ mode guided waves. However, the application of such a technique is possible when the attenuation of such waves is not too high. The determined attenuation values are suitable for ultrasonic testing methods, and the obtained results can be useful for the development of quality control methods of plastic films.
